# Toward safe clinical deployment of remote robotic surgery in Japan: five-year validation of the hinotori™ system using 5G wireless communication

**DOI:** 10.1007/s10147-025-02874-3

**Published:** 2025-10-29

**Authors:** Takuto Hara, Yoshifumi Morihiro, Yuki Horise, Shuhei Komatsu, Masanao Ohashi, Hiroaki Kitatsuji, Akihisa Yao, Yoshihiro Muragaki, Hideaki Miyake

**Affiliations:** 1 Department of Urology, Kobe University Graduate School of Medicine, 7-5-1, Kusunoki-cho, Kobe, 650-0017 Japan; 2 Solution Technology Group, Mobile Innovation Tech Department, NTT DOCOMO, INC., Chiyoda-ku, Tokyo, Japan; 3 System Development Department, Medicaroid Corporation, Chuo-ku, Kobe, Japan; 4 Medicaroid Corporation, Chuo-ku, Kobe, Japan; 5 Faculty of Medical Device Engineering, Kobe University Graduate School of Medicine, Kobe, Japan

**Keywords:** Telesurgery, Robotic surgery, 5G communication

## Abstract

Remote robotic-assisted surgery (RRAS), a form of telesurgery, offers a potential solution to Japan’s surgeon shortage and regional disparities in care. Despite advances in robotic systems and modern communication technologies, including both 5G wireless and wired networks, clinical adoption remains limited due to regulatory, infrastructural, and institutional barriers. This review consolidates five years (2020–2025) of technical and operational validation of the hinotori™ Surgical Robot System—a domestically developed platform—in alignment with the 2022 Japanese Remote Surgery Guidelines. Based on over 30 remote-session evaluations by Kobe University, Medicaroid, and NTT DOCOMO, we summarize system performance across key domains: communication latency, QoS-based prioritization, VPN redundancy, fail-safe mechanisms, electromagnetic compatibility, human–system interaction, and legal compliance. Under optimized Sub6 5G SA conditions, the system consistently achieved a round-trip latency of approximately 100 ms and stable stereoscopic video transmission, even during simulated 1 Gbps congestion. Safety was ensured through automatic standby, dual-cockpit fallback, and real-time monitoring. Although hinotori™ meets technical and safety criteria, full-scale implementation remains constrained by legal requirements—particularly the mandate for an on-site physician under Article 20 of the Medical Practitioners Act. Supervised telesurgery, where remote surgeons assist on-site teams, is legally permissible and may serve as a transitional model. This review integrates technical findings with policy considerations, proposing a path toward safe, equitable, and sustainable RRAS deployment in Japan. To our knowledge, this is the first comprehensive review aligning domestic telesurgical validation with national policy benchmarks, offering a foundation for future regulation, accreditation, and digital surgical integration.

## Introduction

Remote robotic-assisted surgery (RRAS), a form of telesurgery, offers a promising solution to the shortage and uneven distribution of surgeons in Japan, particularly in underserved regions such as rural areas [1]. Despite increasing demand, robotic systems remain concentrated in metropolitan centers. A previous study reported that urban hospitals performed five times more robotic prostatectomies than their regional counterparts [2].

Telesurgery may reduce the disparities between urban and rural areas. However, it is limited by its dependence on ultra-reliable, low-latency communication infrastructure. Simulator and animal studies have shown that round-trip latency beyond approximately 100 ms impairs hand–eye coordination [3, 4]. Furthermore, jitter and packet loss degrade depth perception and compromise operative safety [5, 6]. Based on these findings, the 2022 Japanese Remote Surgery Guidelines (JSS-RSG) stipulated an end-to-end latency of ≤ 100 ms, secure closed-network architecture, and robust redundancy [7].

Globally, several countries have already demonstrated the feasibility of telesurgery under real-world clinical conditions. Europe has recently demonstrated clinical feasibility with the Bordeaux–Beijing partial nephrectomy in 2024, achieving 132 ms latency over 8264 km [8]. In 2025, the United States completed its first telesurgical procedure under formal FDA approval, performing a transcontinental operation between Florida and Angola [9]. China has rapidly implemented dozens of long-distance procedures since 2019, supported by its 5G infrastructure[10] and domestic robotic systems[11]. These cases highlight that clinical-grade telesurgery is achievable when supported by appropriate technical and regulatory frameworks.

Japan has achieved near-universal deployment of fiber-optic infrastructure, which offers high bandwidth, low latency, and strong resistance to electromagnetic interference [12]. These advantages make fiber the gold standard for reliable communication in fixed medical settings. However, high-performance connectivity is not always available at the surgical site, particularly in rural or mid-sized institutions where internal networks may vary. Wired infrastructure is also less suitable for mobile or emergency use. In this context, 5G networks and their wide coverage provide a complementary and flexible option for expanding telesurgical access beyond urban centers.

**Fig. 1:**
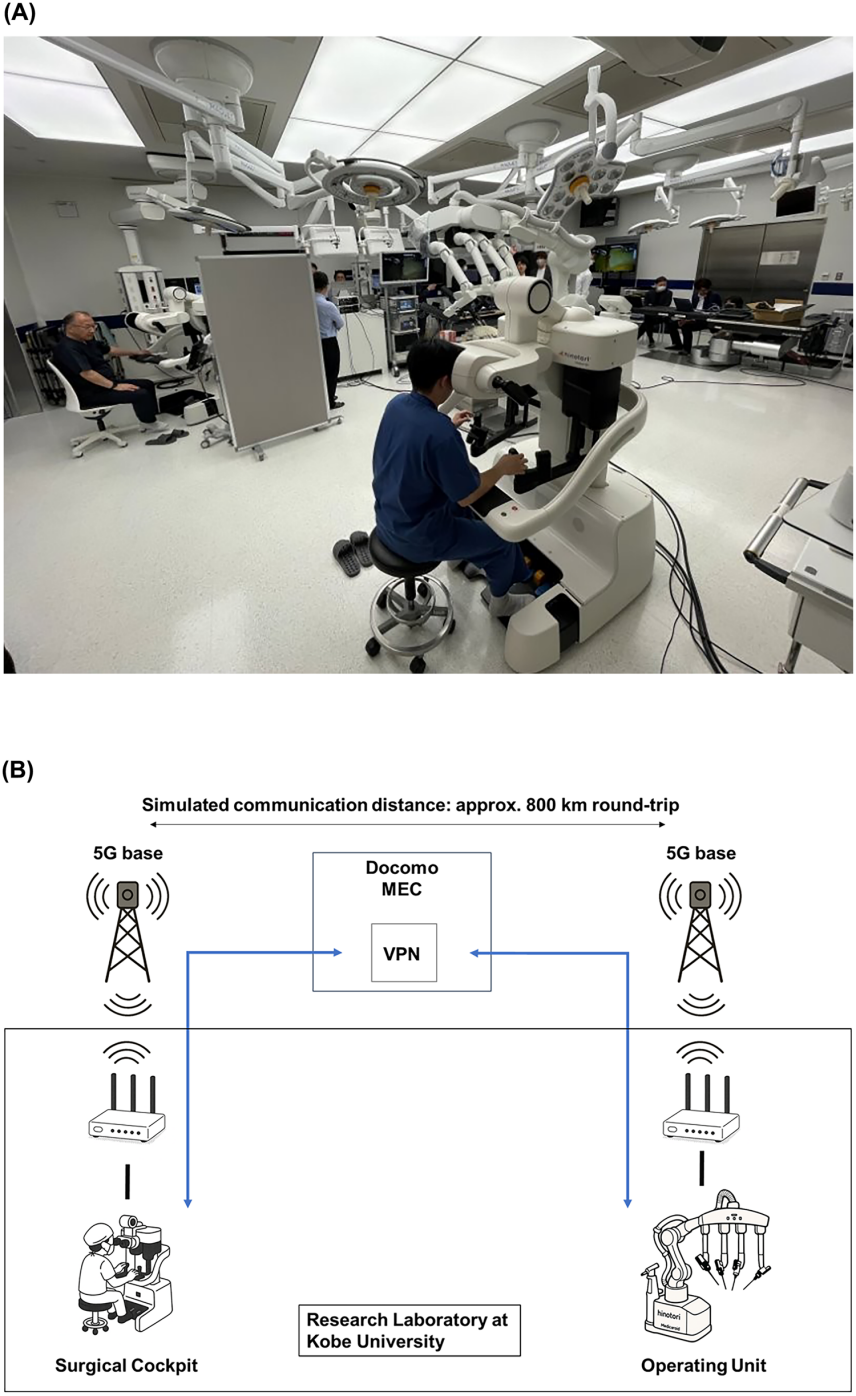
Remote telesurgical validation using the hinotori™ surgical robot system. **A** Photograph from a demonstration experiment simulating remote telesurgical support. Although the master cockpit and the operation unit were located in the same operating room, the system was configured to emulate remote operation conditions. **B** Network configuration used in the validation experiment. 5G wireless access was combined with a wired backbone via multi-access edge computing (MEC) and secured using a virtual private network (VPN). This architecture simulated a round-trip communication distance of approximately 800 km. Over 30 sessions were conducted to evaluate latency, jitter tolerance, failover response, and communication stability

This review consolidates five years (2020–2025) of technical and operational validation of the hinotori™ Surgical Robot Systemin the context of the JSS-RSG that were conducted by Kobe University, Medicaroid, and NTT DOCOMO [9] (Fig. 1). Drawing from over 30 remote-session demonstrations, we summarize system performance in relation to national benchmarks and discuss practical considerations for future clinical deployment. This review highlights selected findings from validation studies, while detailed quantitative analyses are outside its scope and will be addressed in a separate publication.

To our knowledge, this is the first comprehensive review that directly links long-term domestic validation of a commercially available telesurgical platform with Japan’s national regulatory standards. An evidence-based foundation is necessary for future policy, accreditation, and safe clinical rollout.

## Communication and control infrastructure for safe telesurgery

Communication reliability is important for performing telesurgery. Network disruptions, such as latency, jitter, or packet loss, can impair visualization or instrument control during critical moments, directly compromising patient safety [4, 14]. Round-trip latency below 100 ms preserves hand–eye coordination during robotic telesurgery performed by experienced surgeons, whereas delays exceeding 150–200 ms significantly impair surgical precision [15, 16], especially in dual-cockpit settings [17]. To mitigate these risks, encrypted and redundant communication channels are recommended in both the guidelines and academic literature [18–20], including the 2022 JSS-RSG [7].

Bandwidth stability is essential for high-definition stereoscopic video and real-time telemetry [21–23]. Standalone (SA) 5G networks offer low-latency, high-throughput connections with network slicing capabilities that outperform non-SA (NSA) configurations [11, 24]. For example, a ~ 5000 km telesurgery trial in China using SA 5G achieved a median one-way latency of 73 ms and zero packet loss, enabling safe long-distance procedures [11].

To evaluate baseline performance in a realistic 5G environment, we tested Sub6-based SA 5G networks [25], which consistently achieved round-trip latency around 100 ms during continuous high-definition video transmission. In contrast, NR-DC configurations [26], which combine Sub6 and millimeter-wave bands, exhibited greater jitter and unstable frame delivery, making Sub6 more suitable for latency-sensitive telesurgical procedures.

To assess communication robustness under realistic mobile network conditions, we collaborated with NTT DOCOMO to simulate bandwidth congestion at a shared 5G base station. In telesurgery, it is important that surgical video and control signals are transmitted without delay, even when many other users are sharing the same network.

In NTT DOCOMO's 5G Wide service, base stations perform quality of service (QoS) control over a radio interface based on 5QI information [27, 28]. This ensures stable performance despite competing traffic. Without QoS control, packet loss and video degradation were observed with background traffic as low as 200 Mbps. On the other hand, with QoS enabled, stable transmission was maintained even under competing traffic exceeding 1 Gbps. These findings underscore the importance of packet prioritization for ensuring telesurgical safety on public networks.

Redundancy was evaluated by configuring two independent communication pathways(a primary and a backup line)and conducting failover testing to ensure uninterrupted telesurgical operation during simulated line failures. This configuration enabled automatic tunnel recovery and dynamic rerouting. Simulated failures, including SA-NSA handovers, showed VPN reconnection within 5–11 s, without the need to reboot the system. Other research groups have demonstrated that combining primary connections with multi-carrier failover mechanisms can further improve network robustness during communication failures [18–20].

In addition to network-level redundancy and prioritization, transport protocol selection also plays a critical role in maintaining low-latency surgical data exchange. Standard TCP/IP introduces latency through congestion control and retransmission mechanisms, which may be unsuitable for real-time surgical telemetry [29, 30]. Newer telesurgical systems are increasingly adopting UDP-based streaming protocols or real-time optimized protocols, such as QUIC, which offer faster transmission and reduced jitter in network environments prone to loss [31, 32].

In summary, when configured with Sub6-based 5G SA, QoS prioritization, and robust VPN redundancy, the hinotori™ Surgical Robot System fulfills essential communication standards for safe and continuous telesurgery.

## Robotic system design for remote operation

While robust networks are essential, telesurgery also requires robotic platforms that can operate safely and responsively over distance. Most existing surgical robots, including da Vinci [33] and hinotori™ [34], were originally designed for on-site cockpit use. Adapting them for remote application necessitates reengineering software architectures to manage control signals and video feedback across wide-area networks without latency or desynchronization [35]. The JSS-RSG mandates that the master–slave configuration must support physical separation of the cockpit and patient unit [7]. However, progress has been slow due to regulatory caution and limited commercial incentive to redesign FDA-cleared robots [36].

Among the required elements, visual feedback is important because surgeons rely on stereoscopic 3D imagery to perceive depth, identify tissue planes, and perform precise movements [37]. Any degradation in this visual information, such as reduced frame rate, compression artifacts, or narrowed field of view, can impair detection of microbleeds or subtle anatomical landmarks [13, 38–40]. The JSS-RSG requires high-resolution stereoscopic video [7]. Multiple studies have reported that 3D imaging improves depth perception, enhances precision, and reduces physical and cognitive fatigue [38–40]. On the other hand, degraded or delayed imagery has been associated with increased error rates and mental strain [44, 45].

In addition to vision, hardware limitations significantly constrain remote autonomy because essential intraoperative functions, such as instrument exchange, lens cleaning, and suction, still require on-site staff. Thus, there is a lack of self-recovery capability in most current systems. Moreover, remote use amplifies the consequences of mechanical failures because the remote surgeon cannot physically intervene. Therefore, functionalities, such as real-time diagnostics, automatic fault detection, and actuator-level redundancy, are necessary [33]. Thus, the JSS-RSG recommends comprehensive preoperative system checks and mandates the presence of on-site technical support [14].

To validate these safety functions under real-world conditions, we conducted structured evaluations of the hinotori™ Surgical Robot System under simulated latency and jitter. Surgeons were able to complete operative tasks even at elevated delay levels, although fine motor accuracy declined as latency increased. These results are consistent with previously reported latency thresholds for safe telesurgical performance [4, 15, 16].

To validate these safety functions under real-world conditions, we conducted structured evaluations of the hinotori™ Surgical Robot System using simulated latency and jitter. Even with delays up to 180 ms, surgeons were able to complete operative tasks without unintended movement or control failure, although fine motor accuracy began to decline beyond 120 ms, which is consistent with latency findings described in Sect. 2 and prior literature [4, 15, 16]. To assess fault recovery and fail-safe mechanisms, we simulated sudden disconnections at both the router and 5G device levels. The hinotori™ robot reliably entered a safe standby mode, halting all instrument motion without erratic behavior, and in most cases, VPN reconnection restored full control without requiring a system reboot.

These recovery mechanisms mirror fault-tolerant designs seen in teleoperation systems used in hazardous environments [46]. Thus, the hinotori™ system fulfills essential safety and resilience criteria. Based on these validations, the hinotori™ robot satisfies core technical requirements for remote telesurgical use, including reliable master–slave separation, high-fidelity stereoscopic vision, automatic fail-safe behavior, and autonomous reconnection following disconnection. Broader clinical deployment will benefit from continued system improvements, particularly in reducing reliance on local staff, incorporating mechanical redundancy, and enabling multi-cockpit operability to support remote mentoring and distributed surgical collaboration.

## Security and safety

Telesurgery aims to integrate cyberspace with the operating room.　Data security is an important consideration, as any breach or system failure during a procedure could have immediate consequences for patient safety [47, 48]. All transmitted data, including surgical video, robotic control signals, and patient information, must be protected from interception or tampering. The 2022 Japanese telesurgery guidelines mandate the use of VPNs with robust encryption to ensure secure communications [7].

5G Standalone (SA) networks adopt a secure-by-design architecture standardized by 3GPP, including mutual authentication between devices and the core network, SIM-based identity protection, and strong 128-bit encryption for both control- and user-plane traffic [28]. These features offer substantial improvements over legacy wireless networks in terms of confidentiality and protection against impersonation.

However, despite these safeguards, 5G's use of an open wireless medium introduces additional vulnerabilities not typically seen in wired fiber networks. Telesurgery systems may be exposed to radio frequency interference, jamming, or rogue base stations that spoof legitimate signals. Such attacks could degrade connectivity, compromise data integrity, or cause service disruptions. Furthermore, the air interface remains susceptible to denial-of-service (DoS) attacks or protocol-layer exploits that can increase latency or interrupt surgical procedures [49, 50].

To meet these safety demands, we adopted a closed network architecture for all validation trials, utilizing a server-based VPN with multi-access edge computing for direct connectivity, thereby isolating all surgical data, including video, control commands, audio, and telemetry, from the public internet. Dual-layer encryption using IP-VPN and IPsec protocols was applied without introducing perceptible latency, and the configuration complied with Japanese medical data protection standards [51].

We simulated sudden network disconnections to test the system’s resilience using a programmable emulator. In each case, the hinotori™ robot entered a predefined safe standby mode, halting all instrument motion immediately and without unintended activity. This confirmed that the software’s fail-safe logic functioned correctly under transient fault conditions. In most scenarios, VPN reconnection restored full control without requiring system reboot, and in dual-cockpit configurations, local operators could assume control seamlessly, enabling uninterrupted surgical continuity.

Electromagnetic interference (EMI) tests were conducted in line with IEC 60601-1-2 [52]. The hinotori™ system was exposed to simulated emissions resembling those from electrosurgical units and radiologic systems. No signal degradation or operational anomalies were observed, except for transient and reversible abnormalities in the video transmission equipment. Our findings are supported by a 2002 survey on EMI by the Ministry of Internal Affairs and Communications [53] that reported no interference across 4G and 5G SA frequency bands. Thus, this suggests hospital infrastructure compatibility.

Real-time system monitoring was implemented using Zabbix [54] to oversee VPN routers, switches, and encoder/decoder systems. Although internal robotic subsystems were not directly monitored due to segmentation, the setup enabled early detection of packet loss spikes or frame delays, allowing timely interventions to prevent disruption. On-site human redundancy, consisting of licensed medical and technical staff that could intervene in the event of system failure, was also maintained throughout all sessions in accordance with Article 20 of the Medical Practitioners Act [55] and JSS-RSG requirements [7]. Simulated blackouts confirmed that control could be transferred safely to the local team, and the robot resumed normal operation once the VPN connection was restored.

In summary, secure architecture, fail-safe logic, EMI resilience, active monitoring, and human redundancy function as an integrated safety system. Fail-safe logic and automatic standby mechanisms provide a passive safeguard against critical faults [56, 57], and real-time monitoring systems actively detect anomalies before they escalate [33]. On-site human redundancy and dual-cockpit configurations further ensure procedural continuity during unexpected disruptions.

## Human–system interaction

Telesurgery introduces unique challenges in human-system interaction due to the physical separation of the surgeon, patient, and support staff, which disrupts real-time task coordination, physical gesture-based communication, and shared situational awareness. As a result, reliance on visual and audio channels is necessary, and the importance of stereoscopic 3D imaging is further magnified in remote contexts where surgeons must rely solely on visual input without tactile feedback [37, 41–43]. Any degradation in video quality, such as jitter, latency, or compression artifacts, increases cognitive load [58]. Cognitive ergonomics are important in cockpit interface design and systems that maintain consistent visual feedback are necessary.

Communication with on-site staff is important for patient safety. Unlike traditional surgeries where ambient cues and physical presence aid coordination, telesurgery depends on mediated audio-visual links. Delays or distortion in this channel can impair response timing and shared situational awareness [4, 14]. To address these challenges, telesurgical systems should incorporate low-latency, high-reliability communication tools, as well as standardized protocols for task delegation and emergency handling [59–61]. In addition, support features, such as telestration, OR overview cameras, and shared vital sign displays, are needed to compensate for the lack of physical interaction and reinforce understanding among the medical staff [62–64].

The hinotori™ system integrates dual-cockpit functionality and structured communication workflows. In validation studies, novice surgeons performed more safely and efficiently when mentored remotely by experts. This highlights the importance of human–human collaboration in remote settings [36, 49]. Moreover, existing robotic surgery training often overlooks the effects of network instability. In our simulation-based curriculum, we introduced challenging conditions such as latency, jitter, and blackout. Surgeons adopted adaptive strategies, including deliberate pauses and slower hand speed, to preserve accuracy under degraded feedback [65, 66].

In summary, successful telesurgery is determined by communication infrastructure as well as optimization of human-system integration, including intuitive interface design, real-time collaboration support, and training under realistic network conditions, particularly for novice users and multidisciplinary teams.

## Regulatory and societal challenges

The clinical implementation of telesurgery in Japan is constrained by legal, financial, and institutional limitations, including the prohibition of remote-only surgery under Article 20 of the Medical Practitioners Act [55], unreimbursed infrastructure costs, and the lack of standardized accreditation systems. Current legal interpretations mandate direct physician–patient interaction prior to surgery, effectively prohibiting fully remote telesurgery unless a licensed surgeon is physically present on-site. This view was reaffirmed in the 2022 statement by the Japan Surgical Society, which emphasized the need for legislative reform to permit independent remote surgery [7]. Financial barriers further limit adoption. Although robotic-assisted surgeries are reimbursed under the national health insurance scheme, this framework does not account for remote surgical participation, meaning that hospitals must independently fund high-speed networks, IT redundancy, and personnel training without financial support. Consequently, even hospitals in underserved regions have little incentive to invest in telesurgical infrastructure.

Data security and institutional liability also pose significant barriers. While encryption and VPNs are technical solutions, national guidelines stress the need for formal legal agreements between institutions to define accountability and prevent disputes in case of adverse events [7, 67]. Yet such agreements remain rare due to legal ambiguity. Furthermore, participation in telesurgical trials is limited to accredited institutions with existing robotic programs and experienced operators; however, Japan currently lacks a unified national framework for certifying telesurgical centers, platforms, and operators. This regulatory gap complicates quality assurance and hinders equitable expansion [7].

Geographic disparities highlight the importance of public support. A few government-funded pilot programs have helped rural hospitals upgrade communication infrastructure; however, there is still no national policy to subsidize ongoing costs such as 5G data usage, equipment maintenance, or staff education. In contrast, other countries, including China [68, 69] and Kuwait [19], have adopted more flexible legal frameworks and introduced reimbursement schemes and public funding to actively promote telesurgery, encouraging both clinical uptake and private sector investment.

To enable sustainable and equitable deployment of telesurgery in Japan, a coordinated strategy is essential. Priority actions include revising outdated statutes, establishing reimbursement mechanisms for remote participation, codifying liability protocols, and implementing a transparent certification and accreditation system. Drawing from successful international precedents, Japan is well-positioned to create a policy framework that safeguards patient safety, encourages technological innovation, and ensures regional equity in surgical care.

## Toward clinical implementation

The next critical phase for implementing RRAS in Japan is a structured transition from technical validation to clinical deployment. The successful implementation of such systems requires technical readiness in addition to supportive regulatory, institutional, and social infrastructure. Engineering validation studies suggest that the hinotori™ Surgical Robot System, when operated under optimized Sub6 5G SA conditions with approximately 100 ms latency, jitter-tolerant buffering, and robust fail-safe mechanisms can safely support telesurgical procedures under real-world network conditions. Initial clinical deployment should begin with investigator-led studies at accredited institutions to confirm surgical safety, system usability, and communication reliability under realistic network conditions.

As discussed previously, legal reform remains a prerequisite for further implementation. Under the current interpretation of Article 20 of the Medical Practitioners Act [53], telesurgery without direct physician–patient contact is not permitted.

**Fig. 2:**
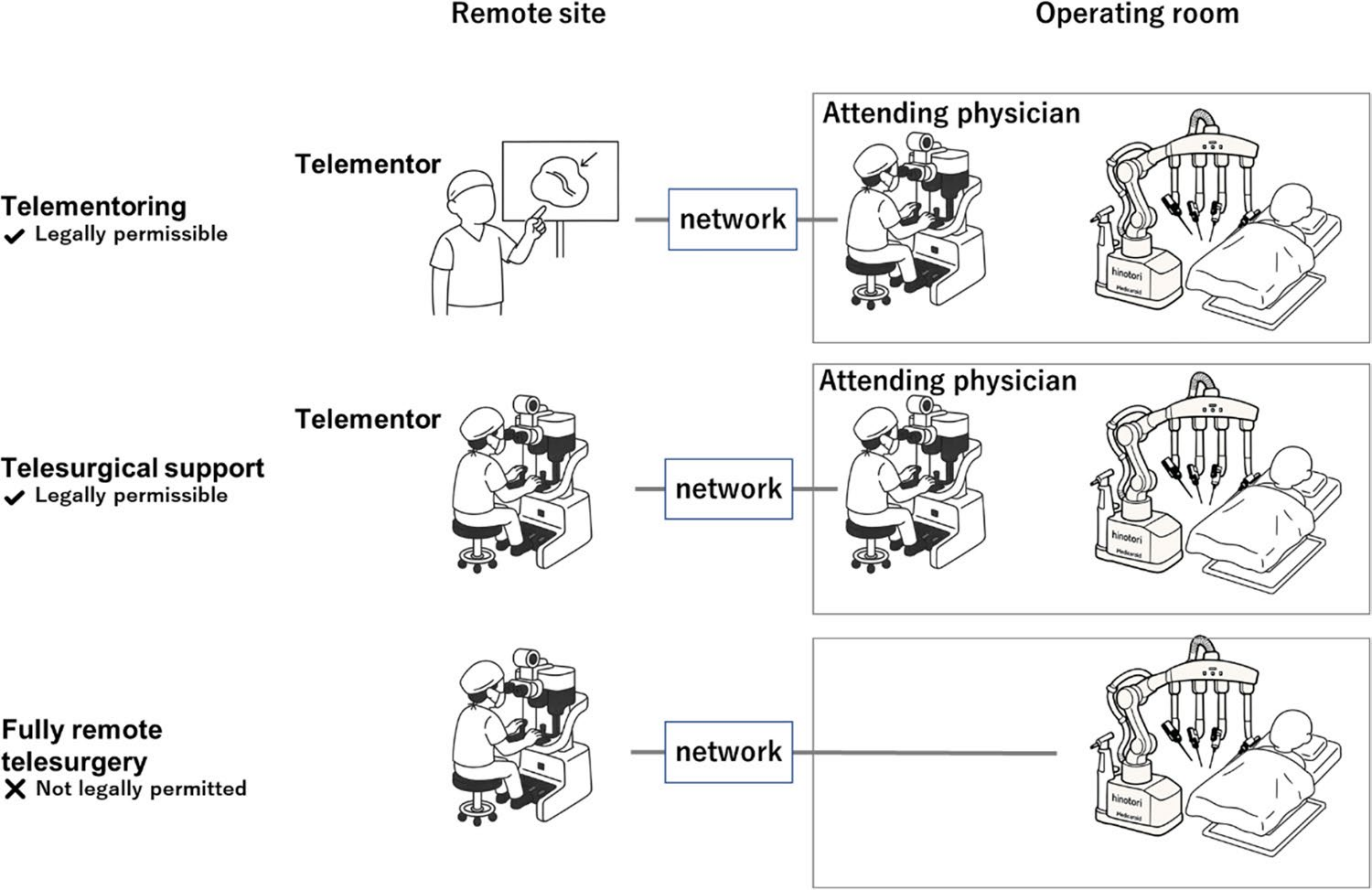
Conceptual models of remote robotic-assisted surgery under current Japanese regulatory conditions. An illustration comparing three models: (top) *Telementoring*, in which a remote expert provides non-invasive visual guidance to an on-site surgeon; (middle) *Telesurgical Support*, where both remote and local surgeons control surgical cockpits collaboratively; and (bottom) *Fully Remote Surgery*, in which the remote surgeon performs the entire procedure without on-site medical staff. Under current Japanese medical law (Article 20 of the Medical Practitioners Act), only telementoring and telesurgical support are legally permissible, whereas fully remote surgery remains restricted

We propose that the near-term objective should focus on supervised remote surgery models, specifically, those in which an on-site licensed surgeon performs the procedure while receiving real-time guidance or support from a remote expert. This approach is already recognized under current Japanese regulations, including the 2022 guidelines issued by the Japan Surgical Society [7], which classify models, such as telementoring or telesurgical support, as being legally compliant (Fig. 2). Domestic validation studies are currently exploring the feasibility of telesurgical support models in which expert surgeons provide remote procedural guidance. Preliminary findings suggest that such systems may enable safe and effective support without compromising surgical quality [70, 71]. Remote guidance enables junior surgeons in underserved areas to receive real-time mentorship from experienced specialists, thereby improving local surgical capacity while maintaining patient safety standards. Supervised remote surgery, thus offering a legally compliant and scalable model that can serve as a foundation for future full telesurgical implementation.

The development of a formal certification system is important. RRAS requires distinct competencies, including latency adaptation, visual compensation, and remote team coordination. Simulation-based training and structured credentialing should be introduced, as well as a national registry for safety monitoring and quality assurance.

In the long term, RRAS could become part of a broader digital surgical ecosystem that integrates telepathology, cloud-based imaging, and remote education. Japan’s robust telecom infrastructure and centralized healthcare policy provide a strong foundation for this transition. Widespread adoption will depend on institutional trust, legal clarity, and sustained public investment. Future policy must prioritize safety, accessibility, and equity as well as technological advancement.

## Declarations

### Registry and the registration no. of the study/trial

N/A.

### Animal studies

N/A.

## References

[1] Takami H, Kodera Y, Eguchi H et al (2024) The shortage of surgeons in Japan: results of an online survey of qualified teaching hospitals that take part in the surgical training programs for board certification by the Japan Surgical Society. Surg Today 54(1):41–52. 10.1007/s00595-023-02697-7 37193795 10.1007/s00595-023-02697-7 PMC10764368

[2] Hara K, Kanda M, Kuwabara H et al (2024) Current status analysis of the prevalence and regional disparities of robot-assisted laparoscopic prostatectomy in Japan using diagnosis procedure combination data. Sci Rep 14(1):24823. 10.1038/s41598-024-75837-9 39438531 10.1038/s41598-024-75837-9 PMC11496839

[3] Moustris G, Tzafestas C, Konstantinidis K (2023) A long distance telesurgical demonstration on robotic surgery phantoms over 5G. Int J Comput Assist Radiol Surg 18:1577–1587. 10.1007/s11548-023-02913-2 37095315 10.1007/s11548-023-02913-2 PMC10124680

[4] Akasaka H, Hakamada K, Morohashi H et al (2022) Impact of the suboptimal communication network environment on telerobotic surgery performance and surgeon fatigue. PLoS ONE 17(6):e0270039. 10.1371/journal.pone.0270039 35709190 10.1371/journal.pone.0270039 PMC9202925

[5] Xu S, Perez M, Yang K et al (2014) Determination of the latency effects on surgical performance and the acceptable latency levels in telesurgery using the dV-Trainer® simulator. Surg Endosc 28(9):2569–2576. 10.1007/s00464-014-3504-z 24671353 10.1007/s00464-014-3504-z

[6] Kim T, Zimmerman PM, Wade MJ et al (2005) The effect of delayed visual feedback on telerobotic surgery. Surg Endosc 19(5):683–686. 10.1007/s00464-004-8926-6 15776211 10.1007/s00464-004-8926-6

[7] Mori M, Hirano S, Hakamada K et al (2024) Clinical practice guidelines for telesurgery 2022: committee for the promotion of remote surgery implementation, Japan Surgical Society. Surg Today 54(8):817–828. 10.1007/s00595-024-02863-5 38829562 10.1007/s00595-024-02863-5 PMC11266380

[8] Fundació Puigvert. Dr. Alberto Breda performs the world’s first transcontinental partial nephrectomy, from Bordeaux to Beijing, via tele surgery [press release]. Barcelona: Fundació Puigvert; 2024.

[9] AdventHealth. Historic telesurgery connects Central Florida and Angola in world-first medical breakthrough [press release]. Central Florida: AdventHealth; 2025.

[10] Qu Y, Zhang Z et al (2019) Preliminary study of the treatment of brain diseases by remote manipulation of deep brain stimulation with 5G communication. Chin J Neurosurg. 35(12):1200–1204

[11] Fan Y, Ma C, Wu X et al (2025) 5G remote robot-assisted hepatobiliary and pancreatic surgery: a report of five cases and a literature review. Int J Med Robot 21(1):e70027. 10.1002/rcs.70027 39744935 10.1002/rcs.70027 PMC11694231

[12] Philpott M, Fellenbaum A, Frey D; Omdia. Global Fiber Development Index: 2020 [Internet]. London (UK): Informa Tech (World Broadband Association); 2020 Oct [cited 2025 Aug 6]. https://worldbroadbandassociation.com/wp-content/uploads/2021/08/FDI-White-Paper-Final_151020.pdf

[13] Horise Y, Aoki Y, Morihiro Y, et al. Future trend: telemedicine using 5G. In: Artificial intelligence in surgery. Singapore: Springer; 2025. p. 199–210. 10.1007/978-981-96-6635-5_15.

[14] Rocco B, Moschovas MC, Saikali S et al (2024) Insights from telesurgery expert conference on recent clinical experience and current status of remote surgery. J Robot Surg 18(1):240. 10.1007/s11701-024-01984-w 38833111 10.1007/s11701-024-01984-w PMC11150305

[15] Sachdeva N, Klopukh M, St Clair R et al (2021) Using conditional generative adversarial networks to reduce the effects of latency in robotic telesurgery. J Robot Surg 15(4):635–641. 10.1007/s11701-020-01149-5. (**2020 Oct 7**) 33025374 10.1007/s11701-020-01149-5

[16] Nankaku A, Tokunaga M, Yonezawa H et al (2022) Maximum acceptable communication delay for the realization of telesurgery. PLoS ONE 17(10):e0274328. 10.1371/journal.pone.0274328 36201429 10.1371/journal.pone.0274328 PMC9536636

[17] Takahashi Y, Hakamada K, Morohashi H et al (2024) Effects of communication delay in the dual console remote robotic surgery system. Surg Today 54(5):496–501. 10.1007/s00595-023-02784-9 38071250 10.1007/s00595-023-02784-9 PMC11026268

[18] Morohashi H, Hakamada K, Kanno T et al (2023) Construction of redundant communications to enhance safety against communication interruptions during robotic remote surgery. Sci Rep 13(1):10831. 10.1038/s41598-023-37730-9 37402741 10.1038/s41598-023-37730-9 PMC10319872

[19] Aldousari S, Almarzouq A, Hassan A et al (2025) The era of telesurgery: insights from ultra-long-distance Asia to Middle East human telesurgery robotic assisted radical prostatectomy. J Robot Surg 19(1):108. 10.1007/s11701-025-02274-9 40064737 10.1007/s11701-025-02274-9 PMC11893634

[20] Wakasa Y, Hakamada K, Morohashi H et al (2024) Ensuring communication redundancy and establishing a telementoring system for robotic telesurgery using multiple communication lines. J Robot Surg 18(1):9. 10.1007/s11701-023-01792-8 38206522 10.1007/s11701-023-01792-8 PMC10784335

[21] Ebihara Y, Oki E, Hirano S et al (2022) Tele-assessment of bandwidth limitation for remote robotics surgery. Surg Today 52(11):1653–1659. 10.1007/s00595-022-02497-5 35546642 10.1007/s00595-022-02497-5 PMC9095415

[22] Golini M (2022) Real-time and high-quality video compression for telesurgery. Politecnico di Milano, Milan

[23] Xu J, Sclabassi RJ, Liu Q, et al. Human perception based video preprocessing for telesurgery. In: Proceedings of the annual international conference of the IEEE Engineering in Medicine and Biology Society (EMBS). 2007;2007:3086–9. 10.1109/IEMBS.2007.4352980. 10.1109/IEMBS.2007.4352980 18002646

[24] Global mobile Suppliers Association. 5G-Standalone April 2025. https://gsacom.com/paper/5g-standalone-april-2025/. Accessed 13 May 2025.

[25] NTT DOCOMO. NTT DOCOMO achieves maximum download speed of 6.6 Gbps by utilizing 5G Standalone architecture and 5G NR-DC technology [Internet]. 2024 Jul 30 [cited 2025 Jun 4]. https://www.docomo.ne.jp/english/info/media_center/pr/2024/0730_01.html

[26] NTT DOCOMO. Deployment of 5G NR-DC technology combining Sub-6 and mmWave bands achieving maximum download speed of 6.6 Gbps in Japan. NTT DOCOMO Technical Report [Internet]. 2024 Aug 1 [cited 2025 Jun 4]. https://journal.ntt.co.jp/wp-content/uploads/2020/08/JN202009067.pdf

[27] Rodday N, Streit K, Rodosek GD, et al. On the usage of DSCP and ECN codepoints in Internet backbone traffic traces for IPv4 and IPv6. In: Proceedings of the 2019 international symposium on networks, computers and communications (ISNCC); 2019; Istanbul, Turkey. p. 1–6. 10.1109/ISNCC.2019.8909187.

[28] ETSI. 5G; System architecture for the 5G System (5GS) (3GPP TS 23.501 version 16.6.0 Release 16). ETSI TS 123 501 V16.6.0. Sophia Antipolis, France: ETSI; 2020.

[29] Malik MH, Brinjikji W (2022) Feasibility of telesurgery in the modern era. Neuroradiol J 35(4):423–426. 10.1177/19714009221083141 35341371 10.1177/19714009221083141 PMC9437503

[30] Parsaei MR, Mohammadi R, Javidan R (2017) A new adaptive traffic engineering method for telesurgery using ACO algorithm over software defined networks. Eur Res Telemed 6(3–4):173–180. 10.1016/j.eurtel.2017.10.003

[31] Xia SB, Lu QS (2021) Development status of telesurgery robotic system. Chin J Traumatol 24(3):144–147. 10.1016/j.cjtee.2021.03.001 33744069 10.1016/j.cjtee.2021.03.001 PMC8173578

[32] Skorin-Kapov L, Matijasevic M (2010) Analysis of QoS requirements for e-health services and mapping to evolved packet system QoS classes. Int J Telemed Appl 2010:628086. 10.1155/2010/628086 20976301 10.1155/2010/628086 PMC2952804

[33] Ballantyne GH, Moll F (2003) The da Vinci telerobotic surgical system: the virtual operative field and telepresence surgery. Surg Clin North Am 83(6):1293–1304. 10.1016/S0039-6109(03)00164-6 14712866 10.1016/S0039-6109(03)00164-6

[34] Miyake H, Fujisawa M (2024) Early experience and future prospects regarding use of newly developed surgical robot system, hinotori, in the field of urologic cancer surgery. Int J Clin Oncol 29(6):640–646. 10.1007/s10147-024-02503-5 38625439 10.1007/s10147-024-02503-5 PMC11130061

[35] Avgousti S, Christoforou EG, Panayides AS et al (2016) Medical telerobotic systems: current status and future trends. Biomed Eng Online 15(1):96. 10.1186/s12938-016-0217-7 27520552 10.1186/s12938-016-0217-7 PMC4983067

[36] Patel V, Saikali S, Moschovas MC et al (2024) Technical and ethical considerations in telesurgery. J Robot Surg 18:40. 10.1007/s11701-023-01797-3 38231309 10.1007/s11701-023-01797-3

[37] Huang T, Li R, Li Y et al (2021) Augmented reality-based autostereoscopic surgical visualization system for telesurgery. Int J Comput Assist Radiol Surg 16(11):1985–1997. 10.1007/s11548-021-02463-5 34363583 10.1007/s11548-021-02463-5

[38] Takahashi Y, Hakamada K, Morohashi H et al (2023) Verification of delay time and image compression thresholds for telesurgery. Asian J Endosc Surg 16(2):255–261 36479621 10.1111/ases.13150

[39] Kumcu A, Vermeulen L, Elprama SA et al (2017) Effect of video lag on laparoscopic surgery: correlation between performance and usability at low latencies. Int J Med Robot. 10.1002/rcs.1758 27373237 10.1002/rcs.1758

[40] Wang Y, Ai Q, Shi T et al (2025) Influence of network latency and bandwidth on robot-assisted laparoscopic telesurgery: a pre-clinical experiment. Chin Med J (Engl) 138(3):325–331. 10.1097/CM9.0000000000003257 39149985 10.1097/CM9.0000000000003257 PMC11771599

[41] Yim C, Lo C, Lau MH et al (2017) Three-dimensional laparoscopy: is it as good as it looks? A review of the literature. Ann Laparosc Endosc Surg 2:131. 10.21037/ales.2017.08.01

[42] Xin G, Liu Y, Xiong Y et al (2022) The use of three-dimensional endoscope in transnasal skull base surgery: a single-center experience from China. Front Surg 9:996290. 10.3389/fsurg.2022.996290 36211263 10.3389/fsurg.2022.996290 PMC9537740

[43] Amiri R, Zwart MJW, Jones LR et al (2024) Surgeon preference and clinical outcome of 3D vision compared to 2D vision in laparoscopic surgery: systematic review and meta-analysis of randomized trials. Ann Surg Open 5(2):e415. 10.1097/AS9.0000000000000415 38911624 10.1097/AS9.0000000000000415 PMC11191999

[44] Hammou D, Krasula L, Bampis C, et al. The effect of viewing distance and display peak luminance—HDR AV1 video streaming quality dataset. In: Proceedings of the 2024 IEEE international conference on quality of multimedia experience (QoMEX); 2024. p. 193–9. 10.1109/QoMEX61742.2024.10598289.

[45] Sakthi M, Kerofsky L, Ravi Kumar V, et al. Impact of video compression artifacts on fisheye camera visual perception tasks. In: Proceedings of the 2024 IEEE/CVF conference on computer vision and pattern recognition workshops (CVPRW); 2024 Jun 17. pp. 1301–10. 10.1109/CVPRW63382.2024.00137.

[46] Dede M, Tosunoglu S (2006) Fault-tolerant teleoperation systems design. Ind Robot Int J 33(5):365–372. 10.1108/01439910610685034

[47] Bonaci T, Herron J, Yusuf T, et al. To make a robot secure: an experimental analysis of cyber security threats against teleoperated surgical robots. arXiv preprint arXiv:1504.04339. 2015.

[48] Alemzadeh H, et al. Systems-theoretic safety assessment of robotic telesurgical systems. In: Koornneef F, van Gulijk C, editors. Computer safety, reliability, and security. SAFECOMP 2014. Lecture notes in computer science, vol 9337. Cham: Springer; 2015. p. 217–231. 10.1007/978-3-319-24255-2_16.

[49] National Institute of Standards and Technology. Potential threat vectors to 5G infrastructure. NIST CSWP 0281; 2021. 10.6028/NIST.CSWP.0281

[50] Shokoor F, Shafik W, Matinkhah SM. Overview of 5G & beyond security. EAI Endorsed Trans IoT [Internet]. 2022 Jun. 27 [cited 2025 Aug. 6];8(30):e2. https://publications.eai.eu/index.php/IoT/article/view/1624

[51] Oki E, Ota M, Nakanoko T et al (2023) Telesurgery and telesurgical support using a double-surgeon console system allowing manipulation from two locations. Surg Endosc 37(8):6071–6078. 10.1007/s00464-023-10061-6 37126192 10.1007/s00464-023-10061-6 PMC10150667

[52] International Electrotechnical Commission (2014) IEC 60601-1-2:2014: Medical electrical equipment—part 1–2: general requirements for basic safety and essential performance—collateral standard: electromagnetic disturbances—requirements and tests, 4th edn. IEC, Geneva

[53] Ministry of Internal Affairs and Communications (MIC). Report on research and study of effects of radio waves on medical equipment, etc. 2002 [cited 2025 Jun 4]. https://www.soumu.go.jp/main_content/000066216.pdf

[54] Zabbix Documentation Team. Monitor a network switch or router with Zabbix. Zabbix Documentation [Internet]. [cited 2025 Jun 4]. https://www.zabbix.com/documentation/current/en/manual/guides/monitor_switch

[55] Ministry of Health, Labour and Welfare, Japan. Medical Practitioners Act. Article 20: prohibition of medical treatment without examination [Internet]. e-Gov Law Search; 1948 [cited 2025 Jun 4]. https://elaws.e-gov.go.jp/document?lawid=323AC0000000201

[56] Lombard B, Céruse P, Schultz P (2017) Surgical robotics: safety, legal, ethical and economic aspects. In: Lescanne E (ed) Robotics and digital guidance in ENT-H&N surgery. Elsevier Masson, Paris, pp 227–234

[57] Iqbal S, Farooq S, Shahzad K et al (2019) SecureSurgiNET: a framework for ensuring security in telesurgery. Int J Distrib Sens Netw. 10.1177/1550147719873811

[58] Sasaki F, Ben Naila C, Okada H, et al. Robust video transmission for robot teleoperation over networks with packet loss. In: Proceedings of the IECON 2023 - 49th annual conference of the IEEE industrial electronics society. Singapore; 2023. p. 1–6. 10.1109/IECON51785.2023.10312023.

[59] Clanahan JM, Awad MM (2023) How does robotic-assisted surgery change OR safety culture? AMA J Ethics 25(8):E615-623. 10.1001/amajethics.2023.615 37535506 10.1001/amajethics.2023.615

[60] Léchappé A, Chollet M, Rigaud J, et al. Assessment of situation awareness during robotic surgery using multimodal data. In: Proceedings of the 2020 ACM international conference on multimedia retrieval; 2020; Dublin, Ireland. p. 412–6. 10.1145/3395035.3425205.

[61] Bauernschmitt R, Braun E, Buss M, et al. On the role of multimodal communication in telesurgery systems. In: Proceedings of the 2009 IEEE international workshop on multimedia signal processing (MMSP '09); 2009 Nov 7; Rio de Janeiro, Brazil. 10.1109/MMSP.2009.5293341.

[62] Rudiman R, Mirbagheri A, Candrawinata VS (2023) Assessment of robotic telesurgery system among surgeons: a single-center study. J Robot Surg 17(6):2757–2761. 10.1007/s11701-023-01709-5 37710051 10.1007/s11701-023-01709-5 PMC10678790

[63] Jin ML, Brown MM, Patwa D et al (2021) Telemedicine, telementoring, and telesurgery for surgical practices. Curr Probl Surg 58(12):100986. 10.1016/j.cpsurg.2021.100986 34895561 10.1016/j.cpsurg.2021.100986

[64] Lambert S, Voros S, Canlorbe G, et al. Understanding takeovers and telestration in laparoscopic surgery to inform telementoring system design. In: Proceedings of the 2024 ACM conference on designing interactive systems; 2024; [page range 1–17]. 10.1145/3613904.3641978.

[65] Zhang Y, Wang O, Wang Y et al (2023) Enhancing skill learning with dual-user haptic feedback: insights from a task-specific approach. Front Robot AI 10:1286282. 10.3389/frobt.2023.1286282 38077453 10.3389/frobt.2023.1286282 PMC10701278

[66] Bergholz M, Ferle M, Weber BM (2023) The benefits of haptic feedback in robot assisted surgery and their moderators: a meta-analysis. Sci Rep 13(1):19215. 10.1038/s41598-023-46641-8 37932393 10.1038/s41598-023-46641-8 PMC10628231

[67] Ministry of Health, Labour and Welfare, Japan. Notice regarding the application of Article 20 of the Medical Practitioners Act (No. 0529-4). Tokyo: MHLW; 2022 May 29. Japanese. https://www.mhlw.go.jp/content/10808000/000936160.pdf. [cited 2025 Jun 4].

[68] International Bar Association. Healthcare and telemedicine in the People’s Republic of China: Legal and regulatory survey [Internet]. 2024 [cited 2025 Jun 4]. https://www.ibanet.org/document?id=Healthcare-Telemedicine-Survey-China

[69] Jin X, Ni Y. Prospering telemedicine a reflection of China's rapid internet development. People's Daily Online. 2024 Sep 27. https://en.people.cn/n3/2024/0927/c90000-20224296.html

[70] NTT Corporation, NTT EAST Corporation, Hirosaki University Hospital, Medicaroid Corporation, Kajima Corporation. NTT demonstrates telesurgery support between two distant hospitals using IOWN APN achieving high-precision and stable remote control of surgical support robots and a communication environment as if participants were in the same operating room. Tokyo: NTT Corporation; 2025 Feb 28 [cited 2025 Jun 4]. https://group.ntt/en/newsrelease/2025/02/28/250228a.html

[71] Sysmex Corporation. Commercial 5G‑based demonstration of remote surgical robot assistance succeeded between Tokyo and Kobe [Internet]. Kobe (Japan): Sysmex; 2023 Feb 3 [cited 2025 Aug 6]. https://www.sysmex‑medical‑meets‑technology.com/_ct/17605428

